# Unraveling the Behavior of Intrinsically Disordered Protein c-Myc: A Study Utilizing Gaussian-Accelerated Molecular Dynamics

**DOI:** 10.1021/acsomega.3c05822

**Published:** 2023-12-01

**Authors:** Kavinda Kashi Juliyan Gunasinghe, Taufiq Rahman, Xavier Chee Wezen

**Affiliations:** † Faculty of Engineering, Computing and Science, Swinburne University of Technology Sarawak, Kuching 93350, Malaysia; ‡ Department of Pharmacology, University of Cambridge, Tennis Court Road, Cambridge CB2 1PD, United Kingdom

## Abstract

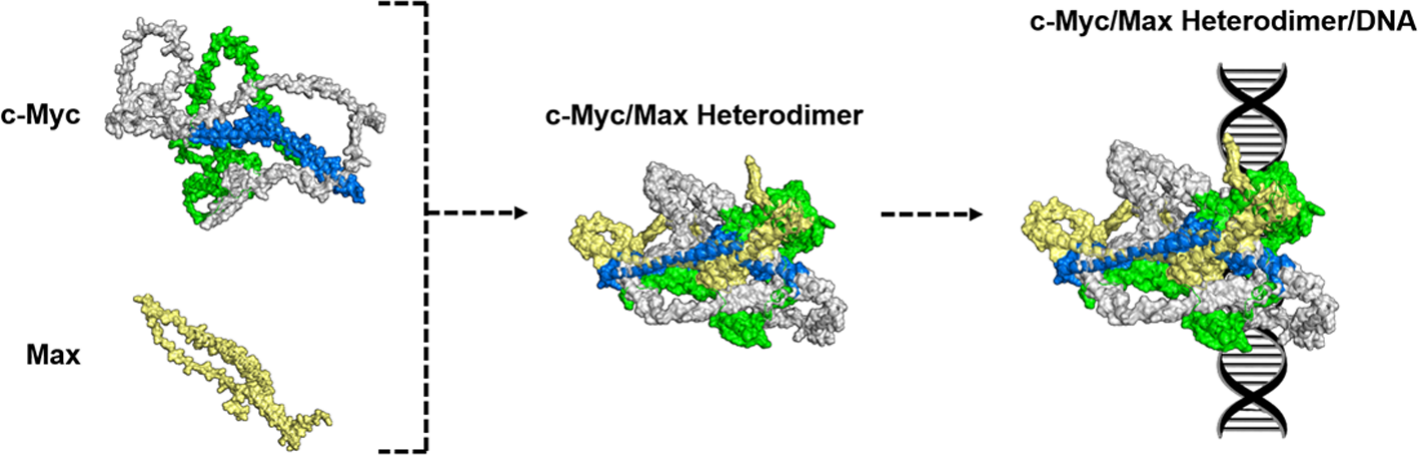

The protein c-Myc is a transcription factor that remains largely intrinsically disordered and is known to be involved in various biological processes and is overexpressed in various cancers, making it an attractive drug target. However, intrinsically disordered proteins such as c-Myc do not show funnel-like basins in their free-energy landscapes; this makes their druggability a challenge. For the first time, we propose a heterodimer model of c-Myc/Max in full length in this work. We used Gaussian-accelerated molecular dynamics (GaMD) simulations to explore the behavior of c-Myc and its various regions, including the transactivation domain (TAD) and the basic helix–loop–helix-leucine-zipper (bHLH-Zipper) motif in three different conformational states: (a) monomeric c-Myc, (b) c-Myc when bound to its partner protein, Max, and (c) when Max was removed after binding. We analyzed the GaMD trajectories using root-mean-square deviation (RMSD), radius of gyration, root-mean-square fluctuation, and free-energy landscape (FEL) calculations to elaborate the behaviors of these regions. The results showed that the monomeric c-Myc structure showed a higher RMSD fluctuation as compared with the c-Myc/Max heterodimer in the bHLH-Zipper motif. This indicated that the bHLH-Zipper motif of c-Myc is more stable when it is bound to Max. The TAD region in both monomeric and Max-bound states showed similar plasticity in terms of RMSD. We also conducted residue decomposition calculations and showed that the c-Myc and Max interaction could be driven mainly by electrostatic interactions and the residues Arg299, Ile403, and Leu420 seemed to play important roles in the interaction. Our work provides insights into the behavior of c-Myc and its regions that could support the development of drugs that target c-Myc and other intrinsically disordered proteins.

## Introduction

Intrinsically disordered proteins (IDPs) are proteins that lack three-dimensional (3D) conformations or secondary structures across the larger part (if not the entire) of their sequence.^1^ Such proteins have been implicated in a myriad of diseases that notably include neurodegenerative diseases, various types of cancers, and cardiovascular diseases.^2^ For example, the misfolding of a well-known IDP, namely, α-synuclein, critically underlies the pathophysiological bases of Parkinson’s disease, while cardiovascular diseases and several tumors (such as those of the breast and ovarian origins) are caused by the overexpression of several IDPs.^3,4^ Under standard physiological conditions, IDPs exist in the form of dynamic structural ensembles that exhibit a high flexibility in their conformations. However, these IDPs can transition from a disordered state to an ordered state when they bind to a partner protein and in some cases to small molecule ligands.^5−8^ However, such a ligand-mediated disorder-to-order transition mostly remains in the local region(s) of the cognate IDP, which still manifests significant amounts of disorder, contributing to their structural plasticity.^9,10^ It is known that approximately 40% of eukaryotic proteins are IDPs, many of which participate in various biological processes, such as protein–nucleic acid recognition (and thus transcriptional regulation), cell signaling, and cell cycle progression.^11^

**Figure 1:**
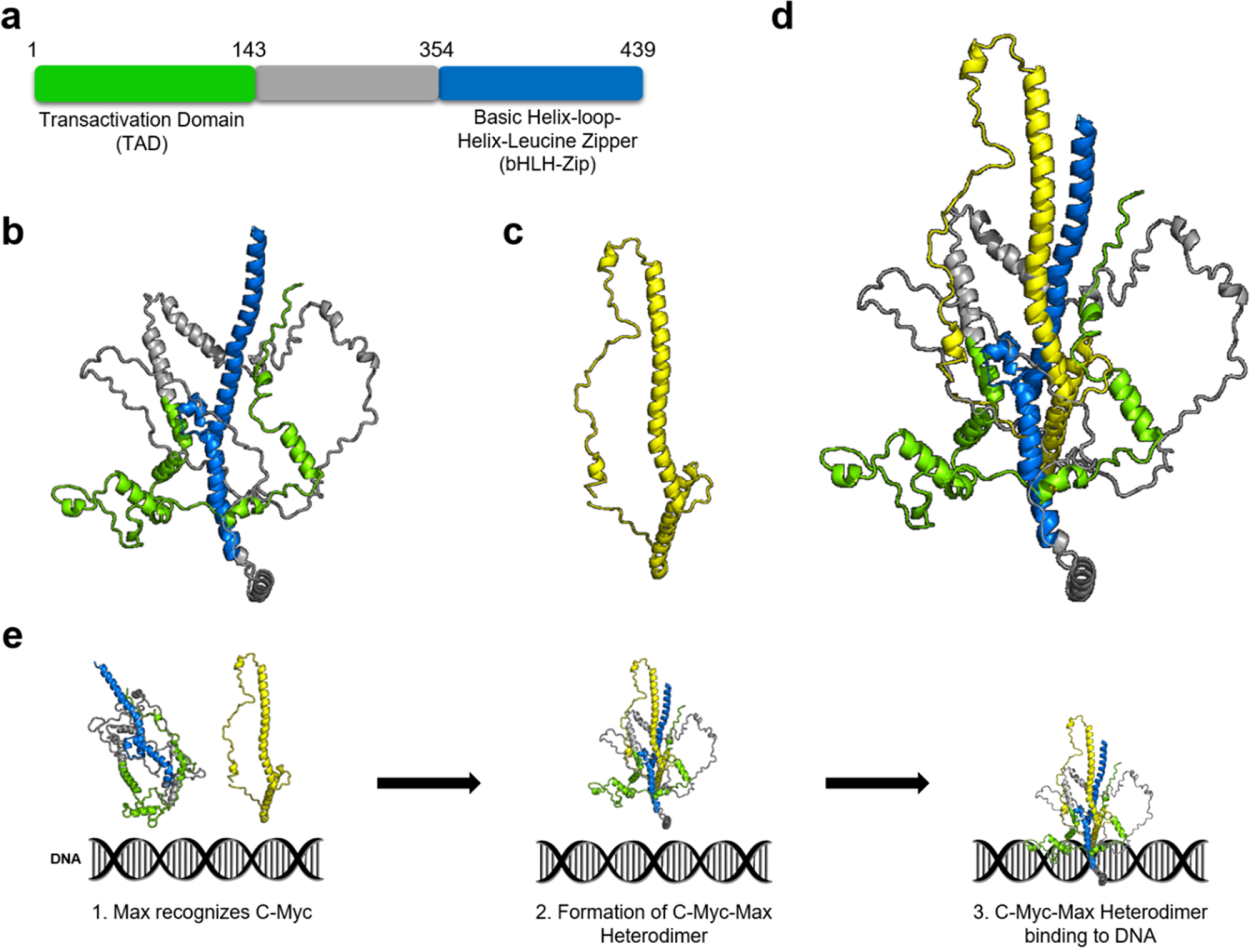
Structure of c-Myc and its interaction with its partner protein, Max. (a) The sequence of c-Myc consists of two important regions, which are the transactivation domain (TAD) region and the bHLH-Zipper motif. (b) The structure of full-length human c-Myc (the initial AlphaFold model is shown in Figure S1). The TAD and the bHLH-Zipper motif are colored green and blue, respectively. (c) The structure of the binding partner, Max. (d) c-Myc and Max bind to the bHLH-Zipper motif of c-Myc to form the c-Myc/Max heterodimer. (e) The c-Myc and Max interaction results in the heterodimer binding to DNA. The structures of c-Myc and Max proteins shown are speculative.

Among the IDPs, c-Myc is noteworthy, being involved in many biological processes such as cell proliferation, adhesion, metabolism, and apoptosis by acting as a regulator of gene expression.^12−14^ The IDP c-Myc belongs to the family of proteins known as the basic helix–loop–helix-leucine zipper (bHLH-Zipper) family. It consists of two important regions, which are the N-terminal region and house the transactivation domain (TAD) (residues 1–143) (Figure 1a,b) that consists of the Myc Box 1 (MB1 Box) and the Myc Box 2 (MB2 Box), which are involved in the recognition of c-Myc by other proteins. In addition to this, the TAD region is also responsible for the involvement of c-Myc in neoplastic transformations, apoptosis, and differentiation.^15−19^ Further experimental studies have demonstrated that residues 1–88 in the TAD region are primarily responsible for its multiprotein complex-forming ability and it exhibits functional plasticity.^15^ The C-terminal region contains the bHLH-Zipper motif (residues 354–439) (Figure 1a,b), which is important for the DNA binding regulatory activity when forming a heterodimer with its partner Max, which is also an IDP (Figure 1c,d). The bHLH-Zipper motif is partially folded and reaches its most stable 3D structure when interacting with Max to form the heterodimer and subsequent binding to DNA (Figure 1e).^20^

As c-Myc is overexpressed in over 70% of human cancers, it is an attractive therapeutic target in oncology.^21^ Inhibitors such as MYC inhibitor 361 and MYC inhibitor 971 can suppress tumor growth in mouse models, promote immune cell infiltration, and upregulate PD-L1 on tumors.^22^ However, as indicated before, IDPs often show high conformational plasticity and can adopt many conformations in the absence of their binding partners. This poses a challenge to the druggability aspect of c-Myc, given the fact that it can attain multiple conformations in its IDP regions and does not contain distinct binding pockets.^23−25^ However, one method to target c-Myc would be to identify its most stable conformations when coupled with Max.^24^ To determine stable conformations for c-Myc, a strategy that can be implemented is molecular dynamics (MD) simulations.

MD simulation is a computational method that is used to explore conformational spaces for proteins and ligands and is widely used in studying the behavior of IDPs.^26,27^ These *in silico* approaches are well suited for conformational analysis as they can provide insight into the atomic-level detail of IDPs, other proteins, and complex systems that allow for predicting and understanding biological pathways and their activity.^28^ A limitation of using conventional MD is the presence of energy barriers that hinder molecules and residues from transitioning between conformational states. To overcome this limitation and enhance its accuracy, Gaussian-accelerated molecular dynamics (GaMD) can be used. GaMD had been previously used to study the isomerization in proline-rich disordered sequences that had shown improved conformational diversity, highlighting the importance of cis/trans proline isomerization in protein–protein interactions.^27^ GaMD incorporates enhanced sampling methods and allows the exploration of rugged energy landscapes that are typically constrained in conventional MD, making it easier to explore various conformations and transitions of proteins, in particular, IDPs. This provides a more comprehensive analysis of the dynamics of IDPs.^29^ In short, GaMD can accelerate the exploration of phase space without requiring lengthy simulation times and allows capturing a wide range of possible conformations that IDPs can adopt.^30^ In addition to this, GaMD simulations allow millisecond-time-scale events of membrane and globular proteins to be captured in hundreds of nanosecond simulation time scales with low energetic noise during reweighting that can be implemented to IDPs.^30^

Previous MD simulation studies of c-Myc had largely focused on the bHLH-Zipper motif and its interaction with Max and DNA binding.^31^ In this work, we used GaMD simulations to evaluate the behavior of c-Myc and its important regions (TAD; bHLH-Zipper) in its nonbound state (monomeric), as c-Myc/Max heterodimer and also following the removal of Max. To assess the trajectory of each of the aforementioned states of c-Myc, we employed root-mean-square deviation (RMSD), radius of gyration (RGyr), and root-mean-square fluctuation (RMSF). We further analyzed the free-energy landscapes (FELs) of the three aforementioned scenarios to predict potential stable conformations of c-Myc in its monomeric and heterodimer states. As far as we are concerned, this is the first MD simulation study ever done with the full-length c-Myc and c-Myc/Max heterodimers.

## Results and Discussion

### GaMD Simulations

We performed 0.5 μs long all-atom GaMD simulations in triplicate to explore the nature of c-Myc in different conformational states using the *ff99SB* force-field and the general purpose water model (OPC). This combination of both the force-field and the water model had previously shown to improve atomistic simulations of IDPs.^32^

### Max Protein Stabilizes the bHLH-Zipper Motif of c-Myc

First, we assessed the RMSD and the RGyr of the different regions of c-Myc in its monomeric state and when bound to Max (henceforth termed Max-bound c-Myc), namely, the bHLH-Zipper motif and the TAD region.

**Figure 2:**
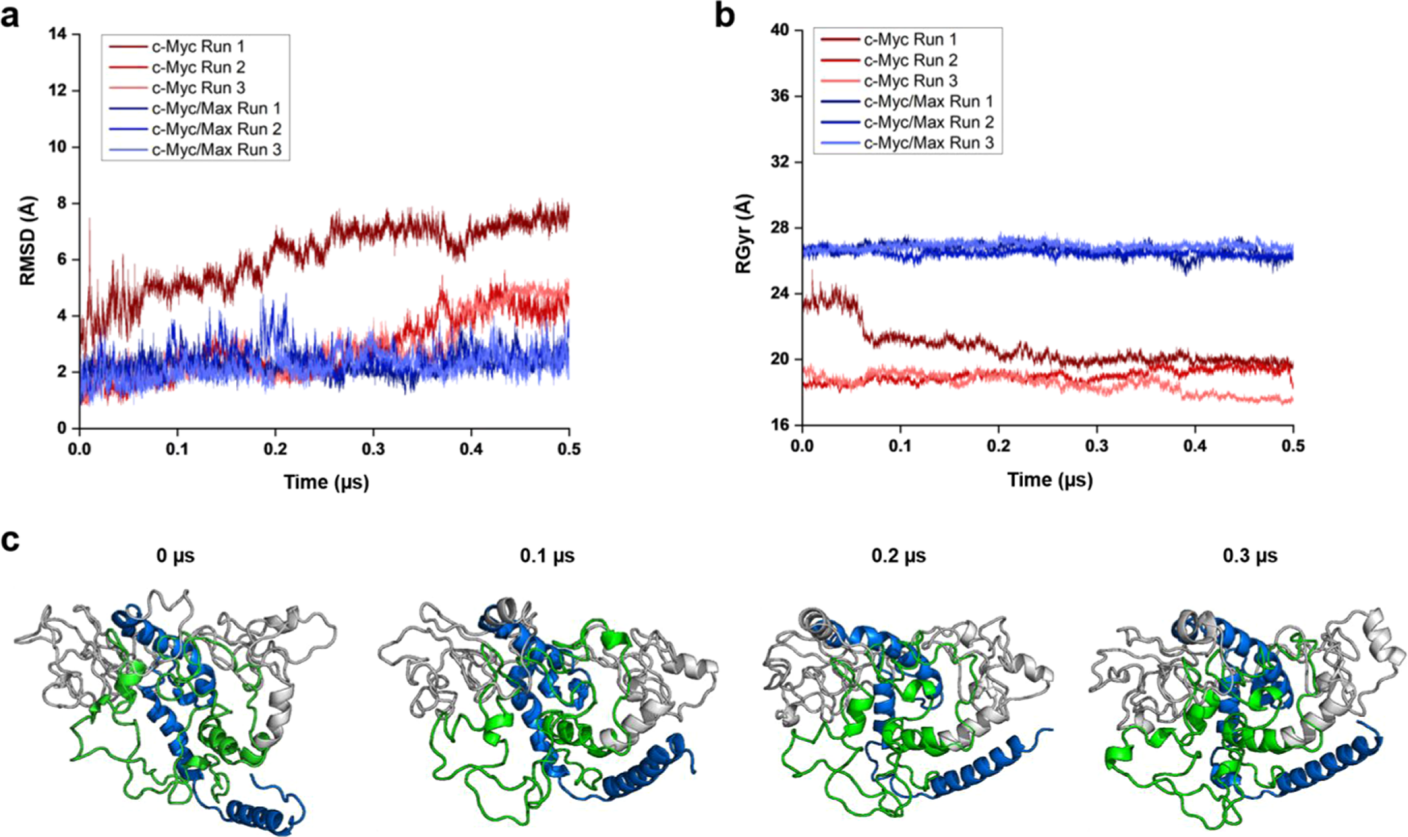
Comparison of (a) RMSD and (b) RGyr of the bHLH-Zipper motif of c-Myc in its monomeric state and Max-bound c-Myc. The c-Myc monomeric state replicates, and the Max-bound c-Myc replicates are shown as red and blue lines, respectively. (c) Time evolution of c-Myc in replicate 1 when in monomeric state up to 0.3 μs shows that bHLH-Zipper increased its compactness over the course of the simulation. The bHLH-Zipper motif and the TAD region are shown in blue and green, respectively.

In all three replicates of monomeric c-Myc, the bHLH-Zipper motif exhibited an RMSD higher than that of Max-bound c-Myc. The three replicates, replicate 1, replicate 2, and replicate 3, showed average RMSDs of 6.13 Å, 3.85, and 3.77 Å, respectively (Figure 2a). Although replicate 2 and replicate 3 exhibited relatively lower RMSDs than replicate 1, they both reached maximum RMSDs of 5.71 and 5.31 Å, respectively. In contrast, the maximum RMSD that replicate 1 achieved was 7.84 Å. The high RMSD in the bHLH-Zipper motif is largely associated with residues between Gln411 and Ala439. Meanwhile, the same motif in the Max-bound state of c-Myc only exhibited minor fluctuations of 2.21 Å, 2.41, and 2.31 Å for replicate 1, replicate 2, and replicate 3, respectively (Figure 2a). In terms of compactness (RGyr), the motif showed an increase in compactness in replicate 1 of c-Myc in its monomeric state from 23.63 to 19.17 Å with a considerable increase at around 0.09 μs (Figure 2b,c). Replicates 2 and 3 also showed a slight increment in the compactness of the motif from 18.43 to 17.97 and 19.34 to 17.17 Å, respectively (Figure 2b). Meanwhile, the motif showed stable compactness in all three replicates when Max is bound to c-Myc, where replicate 1, replicate 2, and replicate 3 showed average RGyrs of 26.67, 26.47, and 26.90 Å, respectively (Figure 2b).

**Figure 3:**
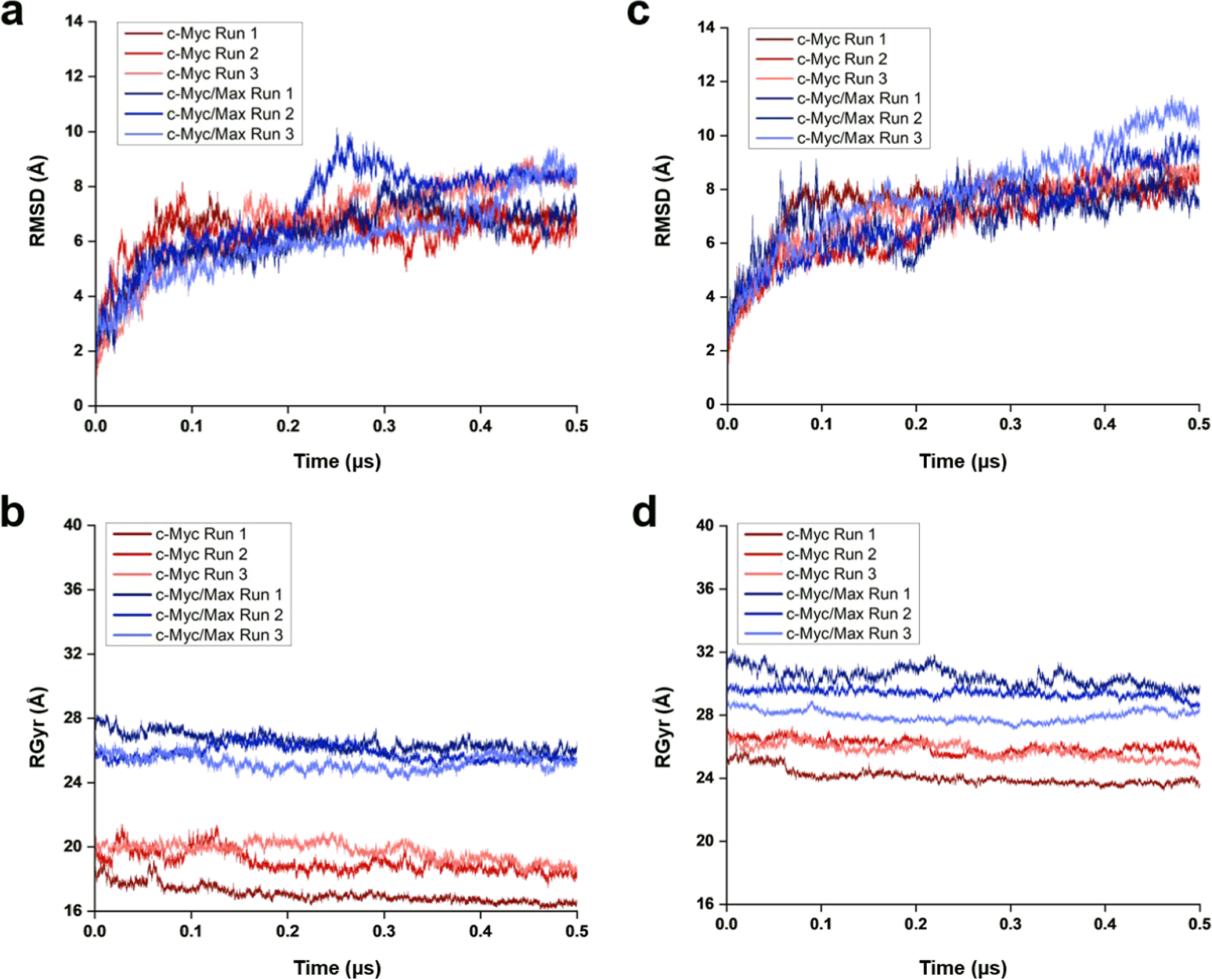
Comparison of RMSD and RGyr of c-Myc in its monomeric state and Max-bound c-Myc. (a) RMSD and (b) RGyr of the TAD region for c-Myc in the monomeric state and Max-bound c-Myc. (c) RMSD and (d) RGyr of c-Myc as a whole in c-Myc in its monomeric state and in Max-bound c-Myc. The c-Myc monomeric state replicates and the Max-bound c-Myc replicates are shown in red and blue lines, respectively. The initial volume of the TAD region was reduced during the modeling of the c-Myc/Max heterodimer.

Next, we analyzed the RMSD and the RGyr of another important region of c-Myc, the TAD region. Interestingly, the RMSDs of both c-Myc in its monomeric state and in the Max-bound state did not stabilize (Figure 3a). In the monomeric c-Myc replicates, the average RMSDs upon reaching the 0.3 μs mark exhibited were 6.96 Å (replicate 1), 6.41 Å (replicate 2), and 7.99 Å (replicate 3) (Figure 3a). Meanwhile, in Max-bound c-Myc, the replicates exhibited RMSDs of 7.02 Å (replicate 1), 8.27 Å (replicate 2), and 7.43 Å (replicate 3) (Figure 3a). The TAD region also showed an increase in compactness in both c-Myc in the monomeric state and in the Max-bound state (Figure 3b). In the monomeric state, the replicates exhibited a reduction of RGyr from 18.32 to 16.10 Å (replicate 1), 19.88 to 17.45 Å (replicate 2), and 20.10 to 18.15 Å (replicate 3) throughout the course of the simulations (Figure 3b). In Max-bound c-Myc, the RGyr decreased from 27.46 to 25.12 Å (replicate 1), 26.14 to 24.78 Å (replicate 3), and 26.29 to 24.06 Å (replicate 3) (Figure 3b). Although the TAD region in both c-Myc in its monomeric state and when bound to Max showed high plasticity, the highly conserved MB1 Box reached stability in all simulations of c-Myc in its monomeric state and Max-bound c-Myc (Figure S2).

Meanwhile, c-Myc as a whole in its monomeric state and, when bound to Max, exhibited high RMSD fluctuations (Figure 3c). The three replicates of c-Myc in its monomeric state showed average RMSDs of 7.44, 6.70, and 7.11 Å for replicate 1, replicate 2, and replicate 3, respectively (Figure 3c). In the Max-bound state, c-Myc showed average RMSDs of 6.87 Å (replicate 1), 7.11 Å (replicate 2), and 7.96 Å (replicate 3) throughout the course of simulation (Figure 3c). This high RMSD is attributed to the high plasticity of the TAD region. As with the TAD region, the overall protein compactness also increased from 25.14 to 23.17 Å (replicate 1), 26.78 to 24.97 Å (replicate 2), and 26.67 to 24.60 Å (replicate 3) for c-Myc in the monomeric state as a whole (Figure 3d). Meanwhile, Max-bound c-Myc showed an increase in its compactness in replicate 1 from 31.70 to 28.72 Å, while replicate 2 and replicate 3 showed an average RGyr of 29.41 and 27.93 Å, respectively (Figure 3d). It is also noteworthy to mention that qualitatively, after the AlphaFold multimer-based modeling as a heterodimer, both c-Myc and its partner Max manifested significant rearrangement of their IDP regions (Figure S3).

Our simulation results hinted that the binding of Max stabilizes the bHLH-Zipper motif of c-Myc. This stability of the bHLH-Zipper motif is possibly important to prevent the proteasomal degradation of c-Myc through ubiquitination by the E3 ubiquitin ligase complex, SCF^Fbw7^β-TrCP.^33^ Meanwhile, we posit that the high plasticity of the TAD region of c-Myc in the Max-bound state is important for its interaction with other protein partners and cofactors that bind to the TAD region.^34^ These protein partners include transformation/transactivation domain-associated protein (TRRAP), TATA-binding protein-interacting protein 48 (TIP48), and SNW1 and P300/CBP interacting protein (SKIP) that are involved in regulating gene expression, chromatin remodeling, and promoting and enhancing transcriptional activity.^35−37^

### Removal of Max Protein from c-Myc Increased the Compactness Significantly in the bHLH-Zipper Motif Than in the TAD Region

**Figure 4:**
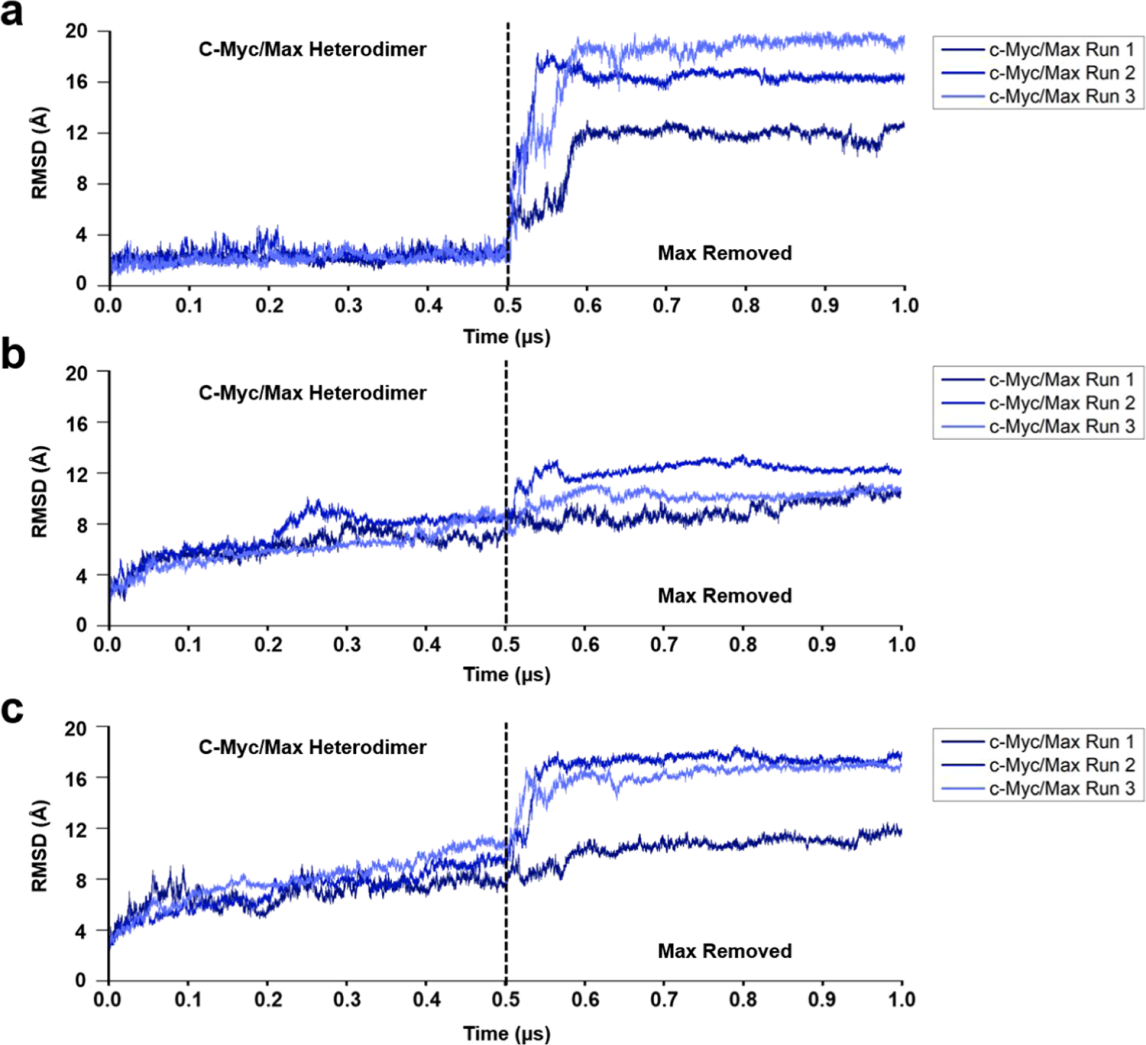
RMSD of c-Myc once Max was removed from the Max-bound state of c-Myc for an additional 0.5 μs. (a) RMSD of the bHLH-Zipper motif when Max was removed from c-Myc. (b) RMSD of the TAD region when Max was removed. (c) RMSD of c-Myc as a whole when Max was removed.

To further analyze the effect that Max could imprint on c-Myc, we removed Max from the Max-bound c-Myc and extended the GaMD simulation of c-Myc alone for an additional 0.5 μs with the three replicates. Based on our RMSD analysis, the removal of Max showed a steep increase in RMSD of the bHLH-zipper motif in all three replicates. In replicate 1, the RMSD increased from an average of 2.21 Å to an average of 11.02 Å post Max removal (Figure 4a). Meanwhile, for replicate 2 and replicate 3, the average RMSD increased by 14.19 Å (RMSD after Max removal: 16.6 Å) and 15.35 Å (RMSD after Max removal: 17.66 Å), respectively, in comparison to when Max was bound (Figure 4a). This larger RMSD shift in the bHLH-Zipper is associated with residues spanning between Pro391 and Ala439.

Next, we investigated the TAD region after the removal of Max. As with the bHLH-Zipper motif, the TAD region also showed an increase in the average RMSD after Max removal. However, in comparison with the bHLH-Zipper motif, the TAD region did not show a significant increase in RMSD (Figure 4b). In replicate 1, the average RMSD increased by 1.97 Å (RMSD after Max removal: 8.99 Å; Figure 4b). In replicates 2 and 3, the average RMSD increased by 3.9 Å (RMSD after Max removal: 12.17 Å) and 2.68 Å (RMSD after Max removal: 10.11 Å), respectively (Figure 4b).

In terms of the overall c-Myc RMSD, in all three replicates post Max removal, a significant increase in RMSD was seen in replicates 2 and 3 (Figure 4c). In replicate 1, the average RMSD increased by 3.62 Å (RMSD post Max removal: 10.49 Å), while replicate 2 and replicate 3 showed an increase in the average RMSD by 9.82 Å (RMSD post Max removal: 16.93 Å) and 8.14 Å (RMSD post Max removal: 16.10 Å), respectively (Figure 4c).

To understand the behavior of c-Myc once Max was removed further, we analyzed the RGyr of the bHLH-Zipper motif, TAD region, and c-Myc as a whole.

**Figure 5:**
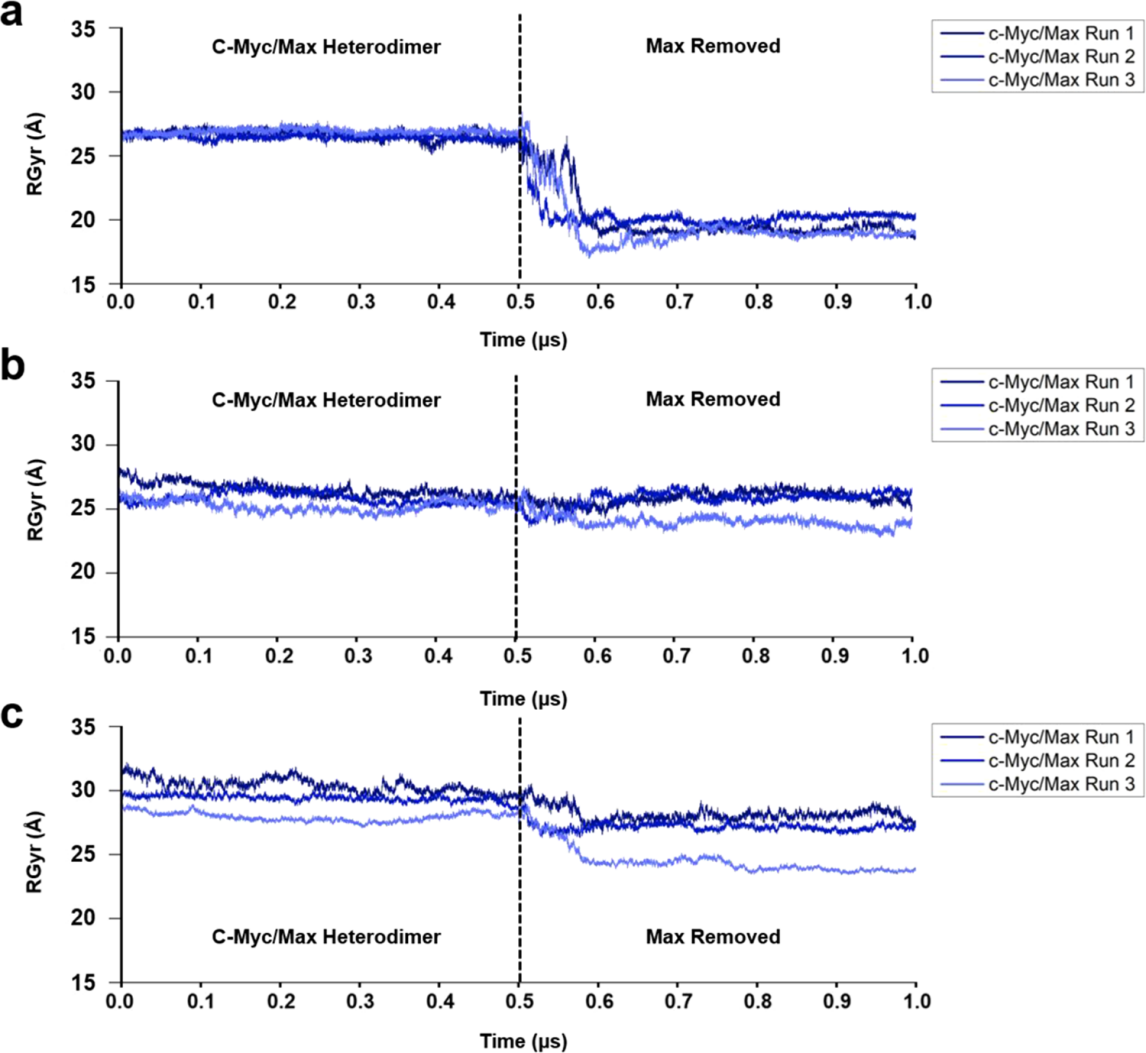
RGyr of c-Myc was removed from Max-bound c-Myc for an additional 0.5 μs. (a) RGyr of the bHLH-Zipper motif when Max was removed from the Max-bound c-Myc. (b) RGyr of the TAD region when Max was removed. (c) RGyr of c-Myc as a whole when Max was removed.

The RGyr analysis demonstrated that the bHLH-Zipper motif underwent significant conformational changes with an increase in compactness. In replicate 1, the RGyr reduced from an average of 26.67 Å to 20.05 Å, increasing its compactness (Figure 5a). Meanwhile, in replicate 2, the stable average RGyr of 26.47 Å reduced to 20.30 Å (Figure 5a). For replicate 3, the RGyr saw a reduction from 26.90 to 19.45 Å (Figure 5a).

Although the bHLH-Zipper motif showed a significant increase in its compactness, the TAD region did not exhibit large changes in its compactness in comparison to the bHLH-Zipper motif (Figure 5b). In replicate 1, replicate 2, and replicate 3, the TAD region RGyr remained stable after the removal of Max.

The protein c-Myc as a whole when Max was removed also exhibited an increase in its compactness in all three replicates. In replicate 1, the RGyr reduced from an average of 30.38 Å to 28.15 Å (Figure 5c). For replicate 2 and replicate 3, the RGyr saw a reduction from 29.41 to 27.51 Å and from 27.93 to 24.54 Å, respectively (Figure 5c).

Our results hinted that the Max protein significantly affects the conformational stability in terms of RGyr and RMSD of the bHLH-Zipper motif, which is important for interaction with DNA.

### Amino Acid Residues from the Disordered Regions of bHLH-Zipper Motif in Monomeric c-Myc Exhibited Higher Fluctuations Than That in Max-Bound c-Myc

Next, we calculated the RMSF for each residue in c-Myc to identify which residues exhibited the highest fluctuations from their initial positions in the two aforementioned scenarios: (a) c-Myc in its monomeric state and (b) Max-bound c-Myc.

**Figure 6:**
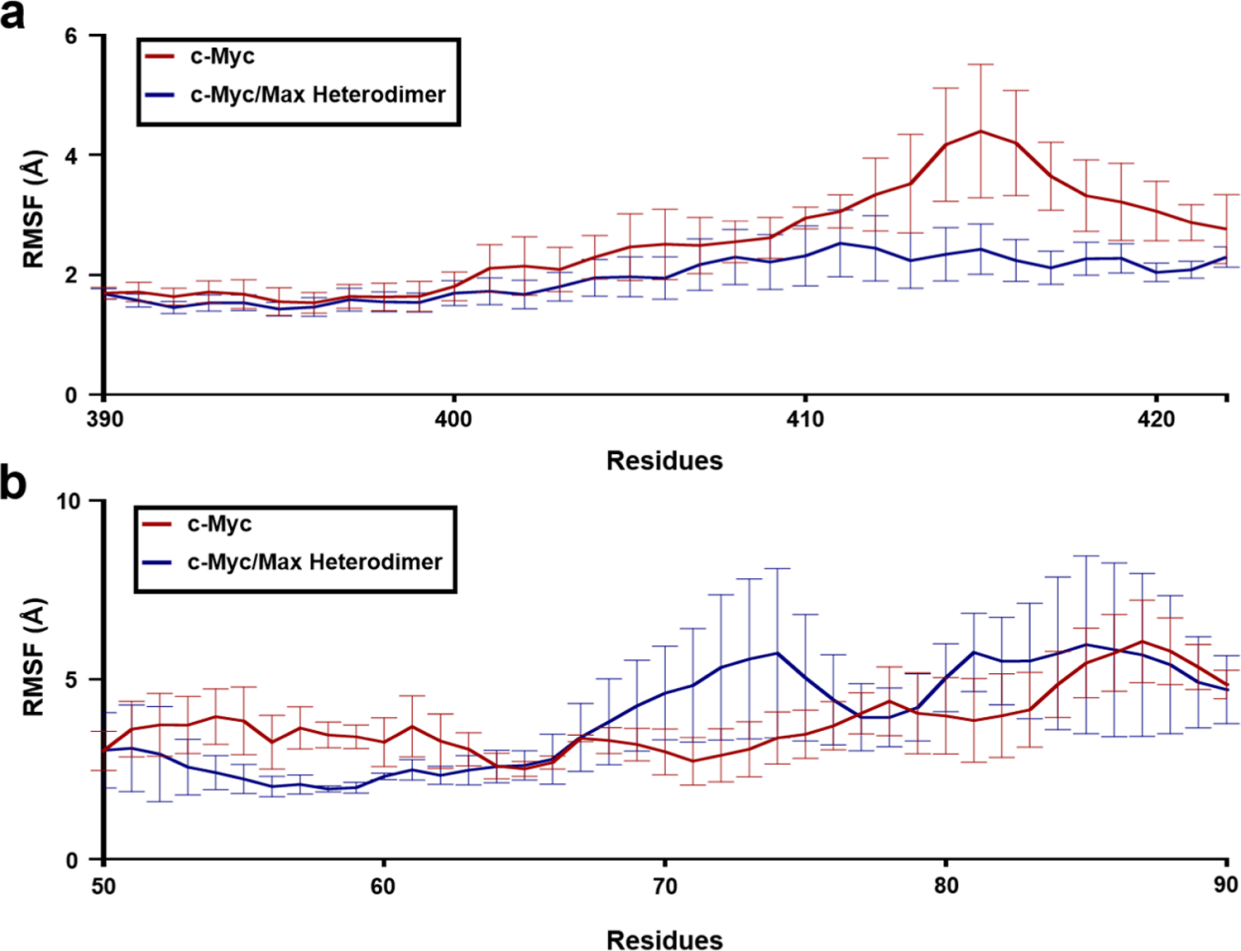
RMSF analysis of the residues of the bHLH-Zipper motif and the TAD region that show variation. (a) The residues of the bHLH-Zipper motif that show variation in RMSF. (b) The residues from the TAD region that show variation in RMSF. The monomeric c-Myc and Max-bound c-Myc are colored red and blue, respectively. The standard errors of mean (SEMs) are shown for all replicates in red bars for monomeric c-Myc and in blue bars for Max-bound c-Myc.

The bHLH-Zipper motif residues spanning between Glu410 and Lys422 showed higher average RMSF in c-Myc in the monomeric state in comparison to c-Myc in the Max-bound state (Figure 6a). The highest RMSF was seen for Ser415 at a mean RMSF of 4.29 Å, with the upper bound being 6.59 Å (Figure 6a).

We also conducted the same analysis on the TAD region. Within the TAD region, the residues that showed higher fluctuation in the c-Myc monomeric state than in Max-bound c-Myc spanned between Trp50 and Arg66 (Figure 6b). These residues represent MB1 Box in c-Myc, which is a highly conserved region. Meanwhile, the residues spanning between Ser67 and Val77 showed higher RMSF in the Max-bound state of c-Myc than in c-Myc in its monomeric state (Figure 6b). These residues belong to the MB2 Box (residues: 28–143), which is less conserved than the MB1 Box. In short, the TAD region did not show variation in RMSF except for the MB2 Box residues (Figure 6b). However, the overall RMSF of the TAD region was greater than the bHLH-Zipper motif in the c-Myc/Max heterodimer. Hence, we speculate that the high fluctuations in the TAD region, particularly in the MB2 Box, are important for interaction between its partner proteins (TRRAP, TIP48, and SKIP).^34−37^

### Combined FEL Plot Revealed Four Conformational States of c-Myc

**Figure 7:**
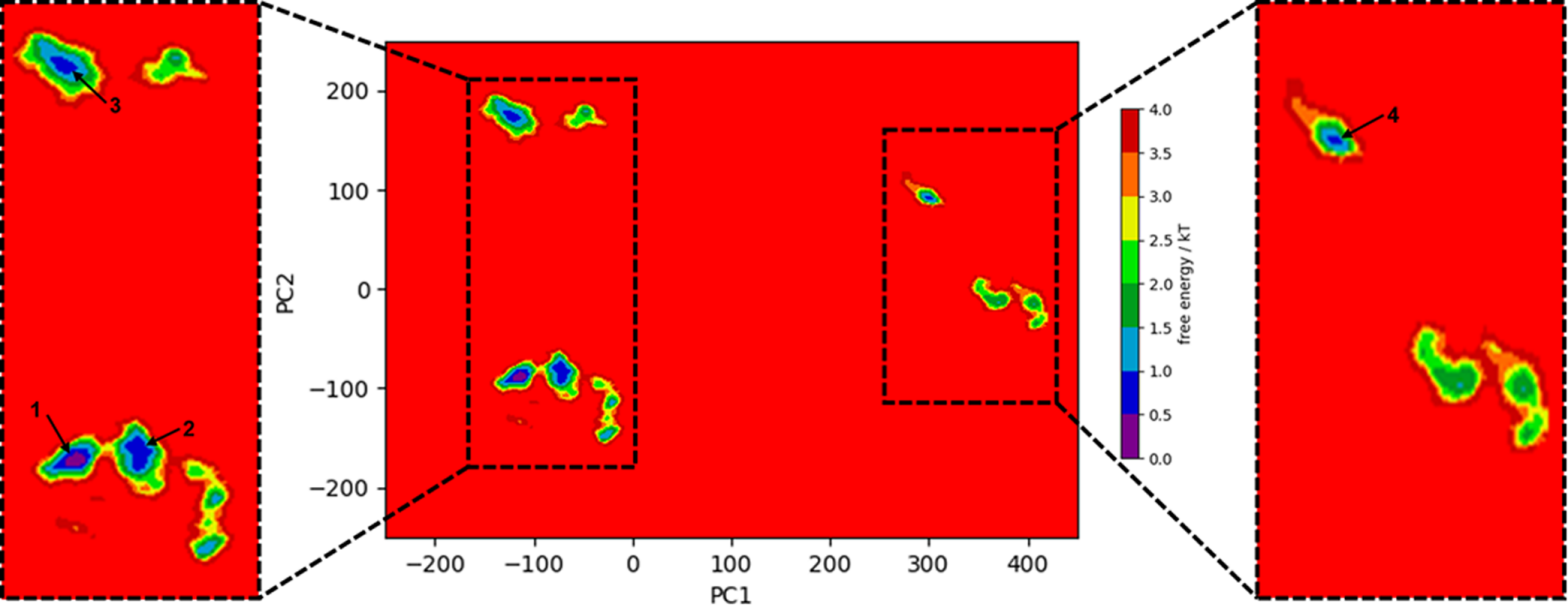
Combined FEL plot of the c-Myc in the monomeric state, Max-bound c-Myc, and when Max was removed. Basins marked as 1, 2, and 3 were achieved with the Max-bound c-Myc replicates. Meanwhile, basin 4 was achieved by replicate 1 of c-Myc in its monomeric state. The lowest energy basin (basin 1) was achieved by replicate 3 of the Max-bound c-Myc simulation.

**Figure 8:**
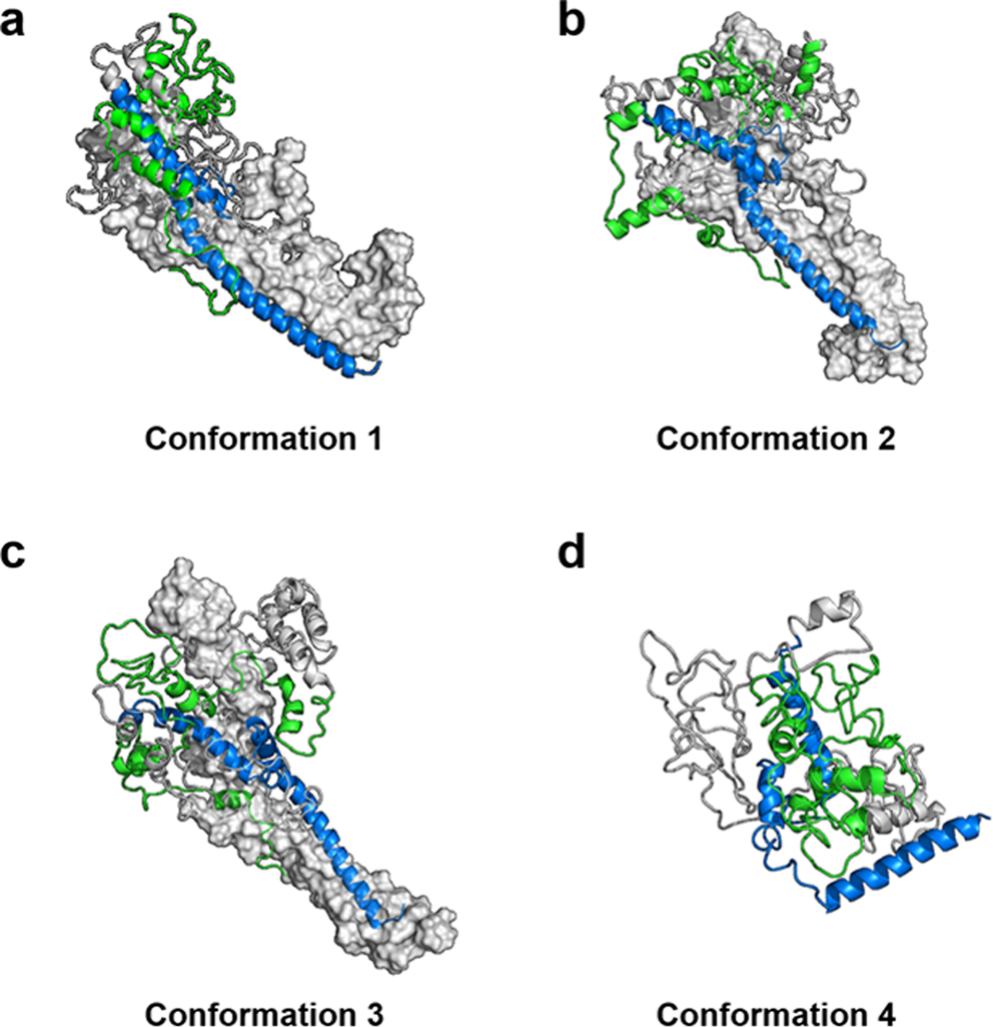
Four conformations correspond to the energy basins of the combined FEL plot. (a) Conformation 1, (b) conformation 2, and (c) conformation 3 were all achieved by the replicates of the Max-bound c-Myc simulations. (d) Conformation 4 of panel (d) was achieved by c-Myc in its monomeric state. The bHLH-Zipper motif and the TAD region are shown in blue and green, respectively. The Max protein is shown as a gray surface.

Next, we generated free-energy landscapes (FELs) for the three systems using all replicates. To recap, the three systems were monomeric c-Myc, Max-bound c-Myc, and Max removed from the c-Myc/Max heterodimer. These FEL plots were used to analyze the dynamic behavior of c-Myc in each case. The combined FEL plot revealed four distinct basins (Figure 7), corresponding to the conformational states achieved by Max-bound c-Myc replicate 3 (conformation 1) (Figures 7 and8a), Max-bound c-Myc replicate 2 (conformation 2) (Figures 7 and8b), Max-bound c-Myc replicate 1 (conformation 3) (Figures 7 and8c), and monomeric c-Myc replicate 1 (conformation 4) (Figures 7 and8d). The conformation that showed the deepest basin was conformation 1 (Figure 8a), with the lowest free energy. This, in turn, showed that c-Myc/Max heterodimerization is important for the conformational stability of c-Myc. The individual FEL plots are shown in Figure S4.

We also opted to compare the structural RMSD between the bHLH-Zipper motif and the TAD region residues between the most stable conformation (conformation 1) and conformation 2, conformation 3, and conformation 4. Between conformation 1 and conformation 2, the bHLH-Zipper motif exhibited an RMSD of 4.38 Å, while the TAD region showed an RMSD of 11.48 Å (Figure 8a,b). Meanwhile, between conformation 1 and conformation 3, the bHLH-Zipper motif and TAD region RMSDs exhibited were 4.73 and 16.48 Å, respectively (Figure 8a,c). Conformation 1 and conformation 4 (c-Myc in the monomeric state) were the largest conformational variations in terms of RMSD, with the bHLH-Zipper motif exhibiting an RMSD of 14.01 Å and the TAD region with an RMSD of 22.28 Å (Figure 8a,d).

These results also hinted that the bHLH-Zipper motif stability is achieved largely with the interaction with the Max protein. However, the TAD region showed a high conformational flexibility between the four conformations. As mentioned before, this flexibility is potentially important for the TAD region to interact with its partner proteins.^34−37^

Next, we compared the residues spanning between Val354 and Ser437 of the bHLH-Zipper motif of the four conformations with the crystal structure of the human c-Myc/Max bHLH-Zip complex (PDB ID: 6G6K) to look for conformational similarity.^38^ Conformation 1 exhibited an RMSD of 3.21 Å when compared to the crystal structure (Figure S5a). Meanwhile, conformation 2 showed an RMSD of 4.11 Å in comparison to the crystal structure (Figure S5b). Both conformation 3 and conformation 4 showed RMSDs of 6.61 and 12.55 Å, respectively (Figure S5c,d). The bHLH-Zipper motif of conformation 1 and conformation 2 and the crystal structure showed good structural similarity. The highest RMSD was attributed to conformation 4, which is achieved in the monomeric c-Myc state.

We also calculated the number of α-helices for conformations 1–4. Notably, we observed the highest number of α-helices for the conformations achieved through c-Myc/Max heterodimerization. These conformations, namely, conformation 1, conformation 2, and conformation 3, exhibited 13, 16, and 14 α-helices, respectively (Figure S6). Conversely, the lowest number of α-helices was seen for conformation 4 (monomeric c-Myc), which showed 11 α-helices (Figure S6).

Our results suggest that the dimerization between c-Myc and Max increases the conformational stability of c-Myc as a whole in terms of the increased number of α-helices in the Max-bound state.

### MM-PBSA Calculations Revealed That the Interaction between c-Myc and Max Is Driven by Electrostatic Interactions

**Figure 9:**
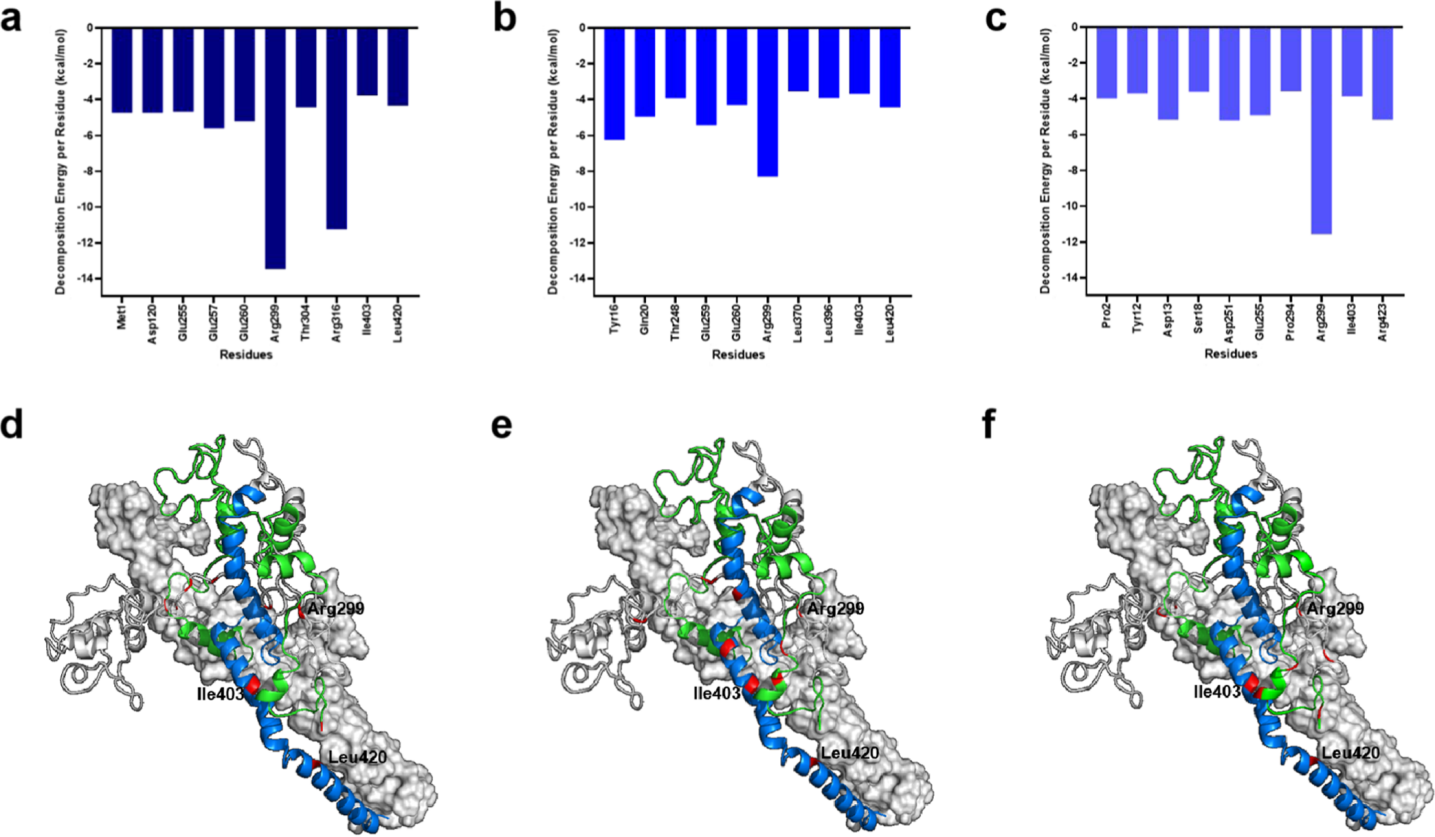
Residues of c-Myc that show high contribution toward binding to Max. (a) The residues that show a high contribution toward binding to Max in replicate 1. (b) The residues that show a high contribution toward the binding of Max in replicate 2. (c) The residues that show a high contribution toward the binding of Max in replicate 3. The residues that are common to all three replicates are Arg299, Ile403, and Leu420. Residues Ile403 and Leu420 belong to the bHLH-Zipper motif of c-Myc. The positions of the residues in (d) replicate 1, (e) replicate 2, and (f) replicate 3 that show significant contribution toward binding to Max are shown in red. The bHLH-Zipper motif and the TAD region are shown in blue and green, respectively. The Max protein is shown as a gray surface.

Next, we calculated the MM-PBSA for the interaction between all residues of c-Myc and Max to determine the driving factor in terms of energy contribution. We observed that this interaction was largely driven by electrostatic interactions (Table 1). This finding aligns with the NMR studies that had demonstrated the important role of electrostatic interactions for c-Myc/Max heterodimerization.^39^ For a detailed understanding of the key binding residues involved in the interaction between c-Myc and Max, we analyzed the decomposition energy per residue of all c-Myc residues. In all three replicates, the highest contribution was seen for Arg299 (Figure 9a–f). Meanwhile, all three replicates also showed high contributions of less than −3 kcal/mol by Ile403 and Leu420 from the bHLH-Zipper motif (Figure 9a–f). All residues that show high contributions (<−3 kcal/mol) in all three replicates are charged residues. The residues that show positive charges are Met1, Pro2, Tyr12, Arg299, Thr304, Arg316, Leu396, Ile403, and Leu420 (Figure S6). Meanwhile, the negatively charged residues include Tyr16, Ser18, Gln20, Asp120, Thr248, Asp251, Glu255, Glu257, Glu260, Pro294, and Leu370 (Figure S7).

**Table 1:** Average Energy Contribution of Each Energy Component in All Three Replicates of Max-Bound c-Myc toward the c-Myc/Max Interaction^a^

	average energy contribution (kcal/mol)		
energy component	replicate 1	replicate 2	replicate 3
Δ*E*_bond_	0.61	0.93	0.70
Δ*E*_angle_	2.07	1.84	2.14
Δ*E*_dihedral_	14.39	15.38	15.48
Δ*E_v_*_dW_	–464.12	–449.59	–481.93
Δ***E***_***EEL***_	**–3202.56**	**–3359.84**	**–3384.90**
Δ*E*_1–4vdW_	3.98	3.10	4.08
Δ*E*_1–4EEL_	83.85	77.47	82.80
Δ*E*_polar_	3243.43	3288.54	3347.16
Δ*E*_nonpolar_	–370.60	–367.74	–376.18
Δ*E*_dispersion_	645.38	644.91	662.85
Δ*G*_gas_	–3561.77	–3710.68	–3761.62
Δ*G*_solvation_	3518.22	3665.70	3633.83
total	–43.55	–144.98	–127.80

a The energy contribution by electrostatic interactions is shown in bold.

**Figure 10:**
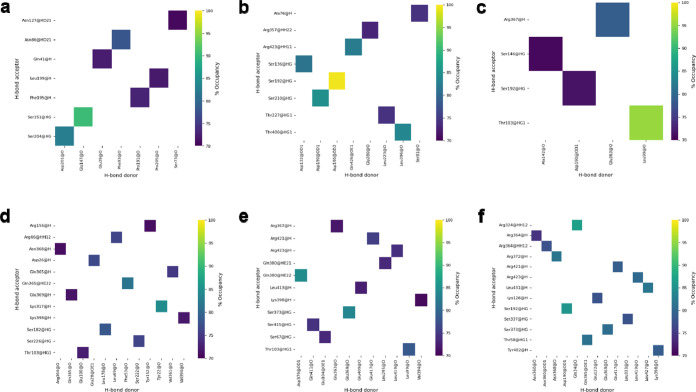
Intramolecular H-bond-forming residues of c-Myc in its monomeric state and the c-Myc/Max heterodimer state. Number of H-bonds formed in the monomeric state in (a) replicate 1, (b) replicate 2, and (c) replicate 3. H-bonds formed within c-Myc when bound to Max in (d) replicate 1, (e) replicate 2, and (f) replicate 3.

Next, we calculated the number of hydrogen bonds (H-bonds) formed within (intramolecular) c-Myc in its monomeric state and when bound to Max, with an occupancy higher than 70%. In replicate 1 of c-Myc in its monomeric state, we observed seven H-bonds, while replicate 2 and replicate 3 showed eight and four H-bonds, respectively (Figure 10a–c). Meanwhile, c-Myc in its Max-bound state showed 12 H-bonds in replicate 1, 11 H-bonds in replicate 2, and 13 H-bonds in replicate 3 (Figure 10d–f). Our H-bond analysis suggested that the number of H-bonds formed within c-Myc upon dimerization with Max increased. Additionally, we calculated the number of H-bonds formed between c-Myc and Max in the heterodimer state (intermolecular). We observed that four H-bonds were formed in replicate 1 of Max-bound c-Myc (Figure S8a). Meanwhile, in replicate 2, we observed three H-bonds (Figure S8b), and in replicate 3, we observed one H-bond formed between the OE2 atom of Glu257 of c-Myc and the NH1 atom of Arg36 of Max.

In relation to our MM-PBSA calculations and H-bond analysis, our results potentially suggest that the c-Myc and Max interaction is largely driven by electrostatic interactions.^40^

### Three Potential Drug Binding Pockets Were Identified within c-Myc in Conformation 1, Conformation 3, and Conformation 4

**Figure 11:**
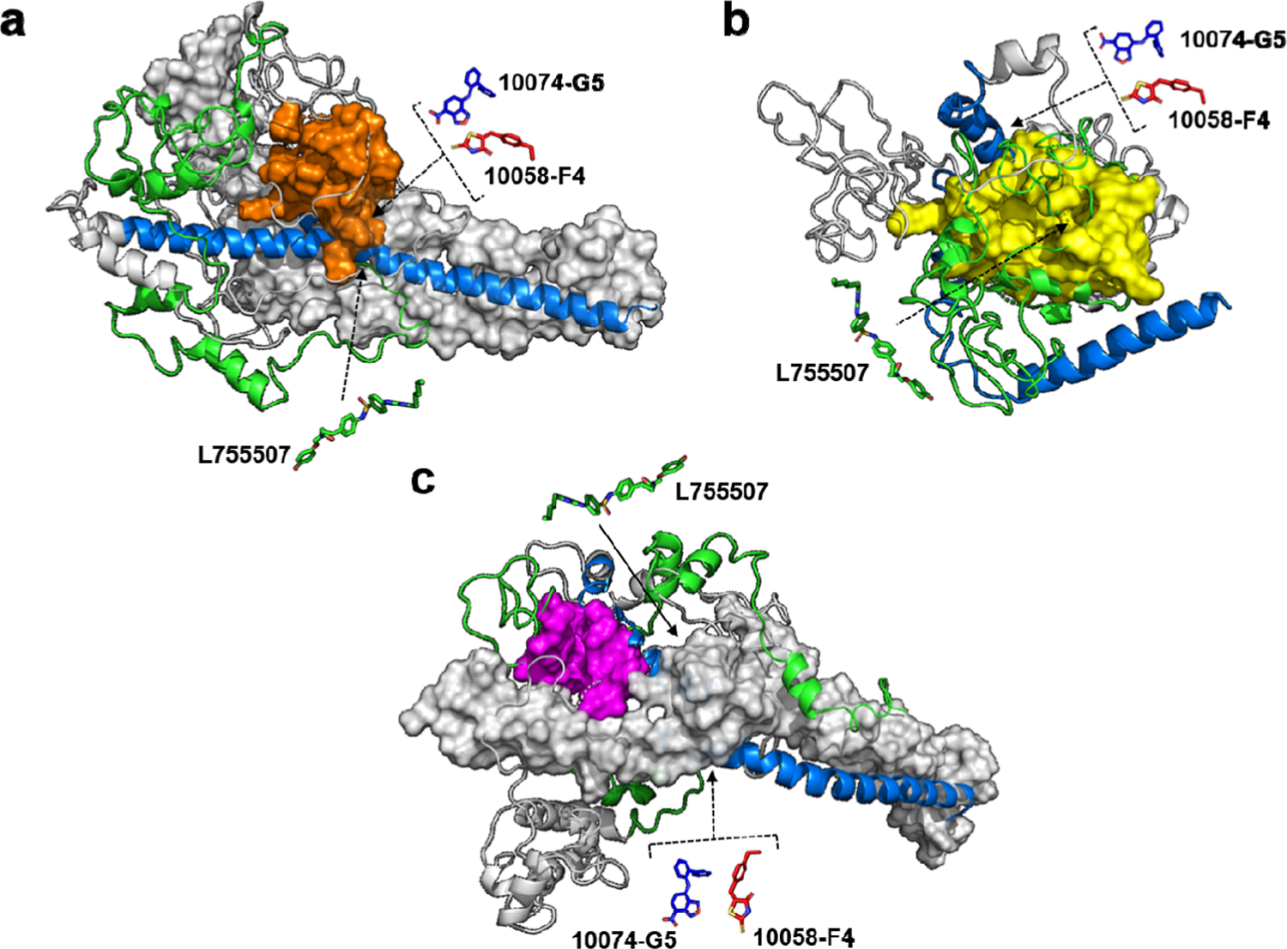
Binding pockets of conformation 1, conformation 4, and conformation 3. (a) The binding pocket 1 is colored orange in conformation 1. (b) Binding pocket 2 is shown in yellow in conformation 4. (c) Binding pocket 3 is shown in pink in conformation 3. The TAD region and the bHLH-Zipper motif are colored green and blue, respectively. The protein surface for Max in panels (a) and (c) is shown as a gray surface. Inhibitors 10058-F4, 10074-G5, and L755507 that bind to the bHLH-Zipper motif region are shown in red, blue, and green, respectively.

As c-Myc is an IDP, determining potential druggable sites is important. Therefore, we attempted to find potential binding pockets within c-Myc in conformation 1, conformation 2, conformation 3, and conformation 4 using the P2Rank Web Server.^41^ We identified three binding pockets with good pocket and probability scores (Table 2). Here, we observed the highest probability and pocket scores for the pocket predicted in conformation 1 (pocket 1), followed by conformation 4 (pocket 2) and conformation 3 (pocket 3) (Table 2 and Figure 11a–c). Notably, all three pockets included residues from the bHLH-Zipper motif that are important for ligand interaction. For example, small molecules such as 10058-F4 and 10074-G5 bind to Glu363-Ile381 in the bHLH-Zipper motif.^42^ Such a binding disrupts the interaction between c-Myc and Max.^42^ Meanwhile, L755507, another inhibitor that is known to block the c-Myc/Max interaction, binds to a pocket within the bHLH-Zipper motif that includes residues between Gln365 and Val406 of c-Myc.^43^ All of these ligand-binding residues were identified in our binding pocket prediction (Table 2). Meanwhile, conformation 2 did not show potential binding pockets with good pocket or probability scores.

**Table 2:** Pockets 1–3 Were Identified in Conformation 1, Conformation 3, and Conformation 4, Respectively^a^

pocket number	residues	**Pocket Score**	**Probability Score**
1	181,187,188,189,196,212,261,272,273,274,275,277,278,279,**383,384,386,387,388,389,390,391,394,398**	20.10	0.85
2	35,36,79,82,105,106,109,112,113,114,115,116,117,118,124,128,131,132,135,138,143,147,148,151,157,194,195,196,197,324,**370,372,373,374,375,376,377,380,393,396**	19.74	0.84
3	63,64,66,70,71,72,74,104,107,108,109,112,114,257,258,260,360,363,364,**367**	16.99	0.80

a The residues are shown as numbers along with the pocket score and the probability score of the binding pockets predicted through the P2RANK Web Server. The residues known to interact with 10058-F4, 10074-G5, and L755507 are shown in bold.

## Conclusions

IDPs, particularly c-Myc, are expressed in many types of cancers, neurodegenerative disorders, and cardiovascular diseases by regulating various cellular processes. Although it is an attractive drug target, its IDP nature poses challenges in druggability. Additionally, simulating the behavior of IDPs is a challenge as they lack a well-defined structure. To address this, we simulated the full-length c-Myc protein in its monomeric state and when interacting with Max using GaMD simulations.

Our study provided additional insights into the behavior of c-Myc in its monomeric states and when Max-bound. The bHLH-Zipper motif showed conformational plasticity in monomeric states rather than in Max-bound c-Myc. In terms of residue fluctuation, the inherently disordered regions of the bHLH-Zipper motif showed higher fluctuations in the monomer states than when c-Myc was bound to Max. Our MM-PBSA calculations and H-bond analysis indicated that the interaction between c-Myc and Max is driven by electrostatic interactions. In terms of druggability, we demonstrated two potential druggable pockets in c-Myc when bound to Max and one pocket in c-Myc in the monomeric state. These binding pockets contain residues from the bHLH-Zipper motif. Our findings provide insights into the nature of conformational changes of c-Myc upon binding to Max and propose this c-Myc/Max heterodimer model for the development of future drug discovery strategies.

## Experimental Methods

### Modeling of Full-Length c-Myc and c-Max Heterodimers

For the monomeric human c-Myc, we used the existing AlphaFold model (https://alphafold.ebi.ac.uk/entry/P01106).

Hitherto, no model of full-length c-Myc and c-Max heterodimer has been available in the literature, though a partial heterodimeric structure of the basic has been solved through X-ray crystallography (PDB: 1NKP).^44^ To model the full-length c-Myc/Max heterodimer, AlphaFold 2.0 multimer was used, which is implemented in ColabFold with its default parameters.^45−47^ This was followed by a brief AMBER-based relaxation of the best-predicted model, which was taken for further MD simulation studies.

### MD Simulations

#### System Preparation

The molecular dynamics (MD) simulations were performed by using the AMBER 20 MD package. All three models were simulated in triplicate. The monomer (c-Myc), the heterodimer (c-Myc/Max), and when Max was removed were parameterized using the *ff99SB* force-field. The excess charges were balanced with Na^+^ ions and solvated with the OPC water model using the *tleap* module.^32^ Long-range electrostatic interactions were treated by using the particle mesh Ewald (PME) method. A nonbonded cutoff distance of 10 Å was used along with a time step of 2 fs for each simulation, and restraints were applied using the SHAKE algorithm.

#### Conventional Molecular Dynamics (cMD) Simulations

The protein c-Myc, in its monomeric state, and the c-Myc/Max heterodimer were minimized in two stages, with the first minimization stage consisting of 500 steps of the steepest descent. This was followed by another 500 conjugate gradient minimizations. The PME cutoff distance and the restraint force were kept at 10 Å and 300 kcal/mol, respectively. Subsequently, heating was carried out for 200 ps, where the temperature was gradually increased from 0 to 300 K along the NVT ensemble followed by 5 ns of equilibration with the NPT ensemble. Next, a production stage cMD was carried out for 50 ns for c-Myc in the monomeric state and Max-bound c-Myc. The preparatory stage was followed by an additional 50 ns of cMD to collect potential statistics (*V*_max_, *V*_min_, *V*_avg_, and σ*V*) required for GaMD simulations.

#### Gaussian-Accelerated Molecular Dynamics (GaMD) Simulations

GaMD equilibration was carried out for 50 ns with a dual boost potential, with both upper and lower deviations of the second potential at 6 kcal/mol. A nonbonded cutoff distance of 10 Å and a time step of 2 fs were used during the simulation. This was followed by a production stage of 500 ns of c-Myc and c-Myc/Max heterodimer and an additional 500 ns for the heterodimer with the removal of Max protein. The temperature was kept at 300 K and monitored using the Langevin thermostat, while the pressure was kept at 1.0 bar and monitored using the Berendsen barostat throughout the simulations.

#### Analysis

The analyses of the simulations and free-energy landscape calculations were calculated using 12500 frames from each simulation using the *cpptraj* module from AMBER 20 and the *pytraj* module, respectively.^48^ MM-PBSA calculations were performed using the *MMPBSA.py* module.^49^ Additionally, the α-helices and binding pocket predictions were done using PyMOL and P2Rank Web server, respectively.^41,50^ The charges of residues were calculated and visualized using the adaptive Poisson–Boltzmann solver (APBS) available in PyMol.^51^

## Data Availability

All molecular dynamics simulations were conducted using the Assisted Model Building with Energy Refinement (AMBER; version 14.0) using a free academic license. Data are available from the corresponding authors on reasonable request.

## Supporting Information Available

(a) The AlphaFold model of c-Myc and the c-Myc/Max heterodimer, (b) the trajectory analyses of the MB1 Box of c-Myc in both monomeric and heterodimer states, (c) the individual free-energy landscapes of c-Myc in monomeric and heterodimer states, (d) the conformation comparisons of the bHLH-Zipper motif of c-Myc and c-Myc/Max heterodimers with the crystal structure (PDB ID 6G6K), (e) the electrostatic map of all residues of c-Myc in its monomeric state, and finally, (f) the hydrogen-bonding patterns of c-Myc/Max heterodimer (PDF)
