# Supplementary material for: Unraveling the Behavior of Intrinsically Disordered Protein c-Myc: A Study Utilizing Gaussian-Accelerated Molecular Dynamics
Source: ACS Omega. 2023 Dec 1;9(2):2250–62. doi: 10.1021/acsomega.3c05822 (PMC10795134; doi:10.1021/acsomega.3c05822)
Supplement: Supplementary file 1 — ao3c05822_si_001.pdf [file ao3c05822_si_001.pdf]

Suppoting Information

Unraveling the behaviour of Intrinsically Disordered Protein c-Myc: A study utilizing Gaussian-Accelerated Molecular Dynamics

Kavinda Kashi Juliyan Gunasinghe[1], Taufiq Rahman[2*], Xavier Chee Wezen[1*]

[1] Faculty of Engineering, Computing and Science, Swinburne University of Technology Sarawak, Malaysia

[2] Department of Pharmacology, University of Cambridge, Tennis Court Road, Cambridge CB2 1PD, United Kingdom

*Corresponding authors: Taufiq Rahman (mtur2@cam.ac.uk), Xavier Chee Wezen (xchee@swinburne.edu.my)

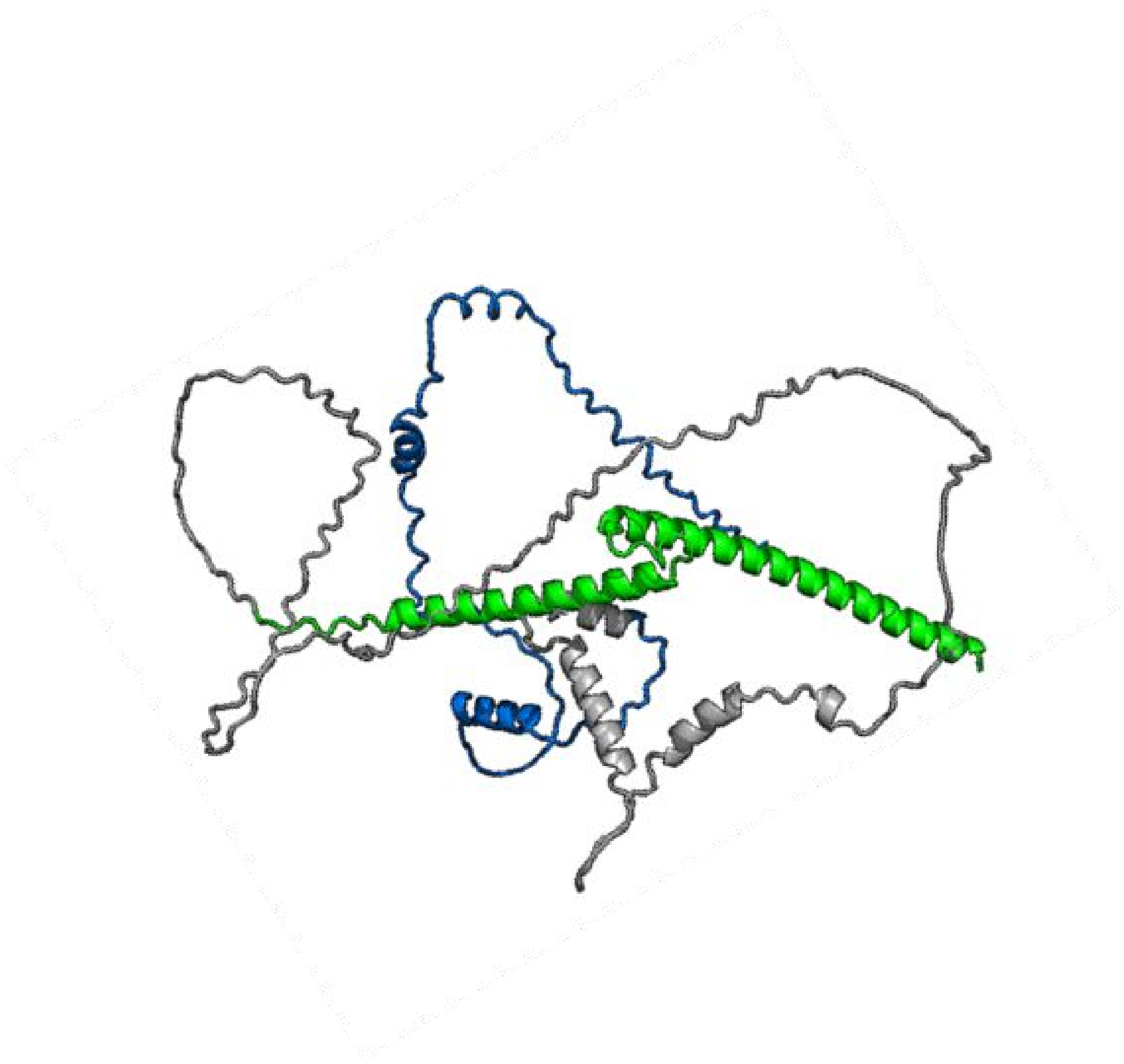

Figure S1. The full length c-Myc structure predicted through AlphaFold (AF-P01106-F1-model_v4). The TAD region and the bHLH-Zipper motif are shown in blue and green, respectively.

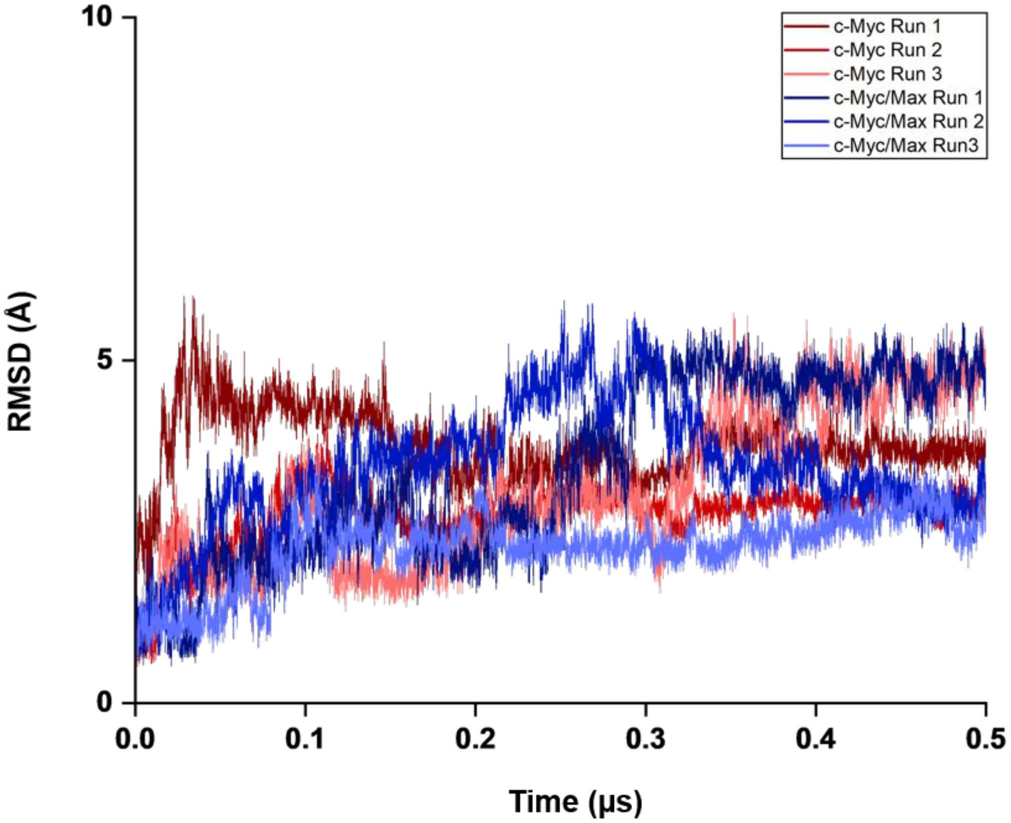

Figure S2. The RMSD of all replicates of c-Myc in its monomeric state and when c-Myc is bound to Max. The RMSD stabilized in all replicates after initial deviations. The protein c-Myc in its monomeric state and when c-Myc is bound to Max are shown as red and blue lines, respectively.

a

b

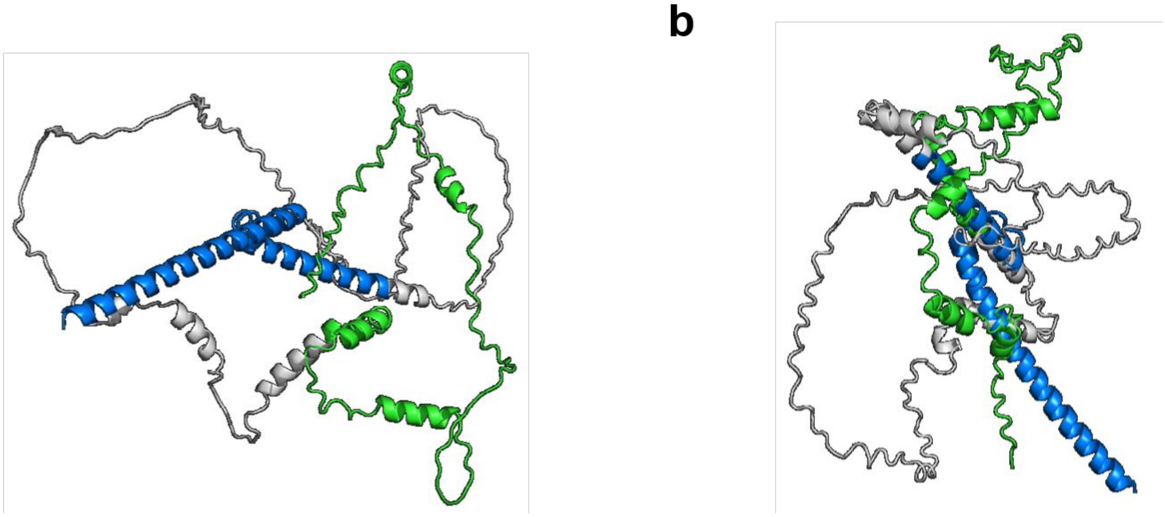

Figure S3. The comparison of structural rearrangement in the bHLH-Zipper motif and the Transactivation Domain (TAD) region of c-Myc upon modeling the c-Myc/Max heterodimer. **(a)** The initial AlphaFold model (AF-P01106-F1-model_v4) of c-Myc. **(b)** The protein c-Myc after c-Myc/Max modeling. The bHLH-Zipper motif and the TAD region are shown in blue and green, respectively.

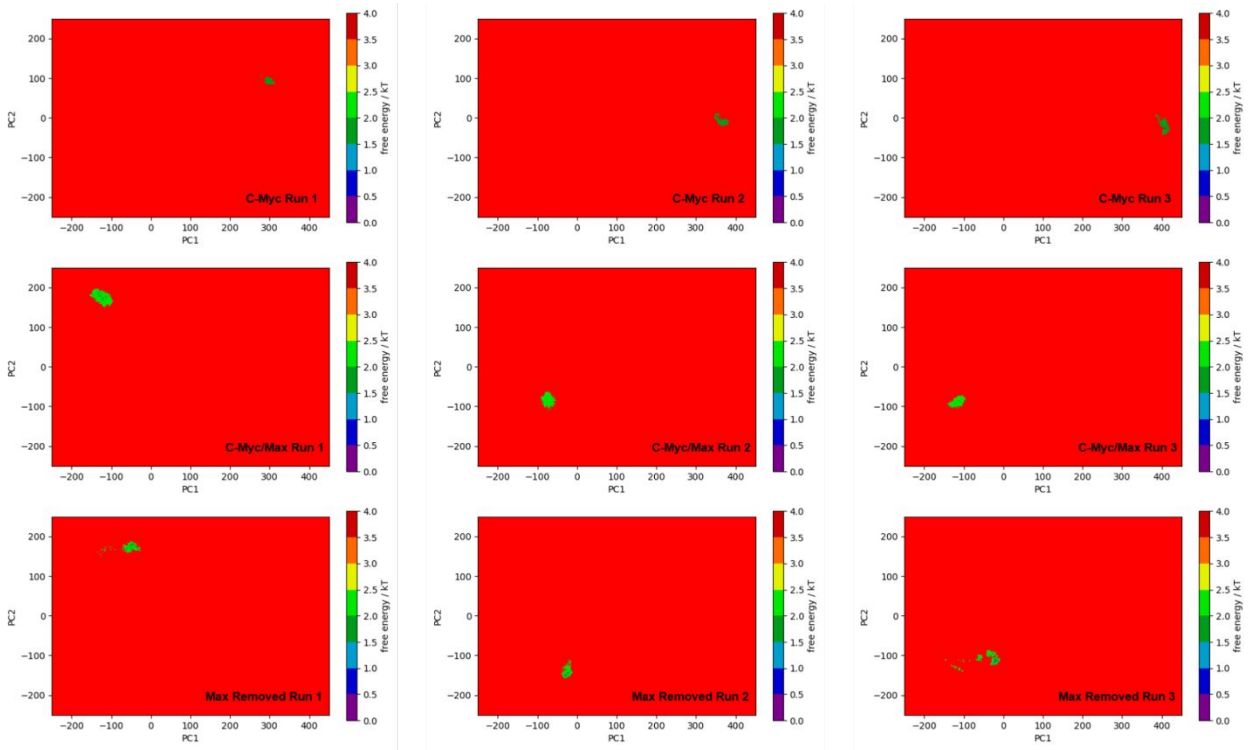

Figure S4. The individual Free Energy Landscape (FEL) plots of c-Myc in its monomeric state, when c-Myc is bound to Max and when Max was removed. A total of 12500 frames were used from each simulation to plot the FEL.

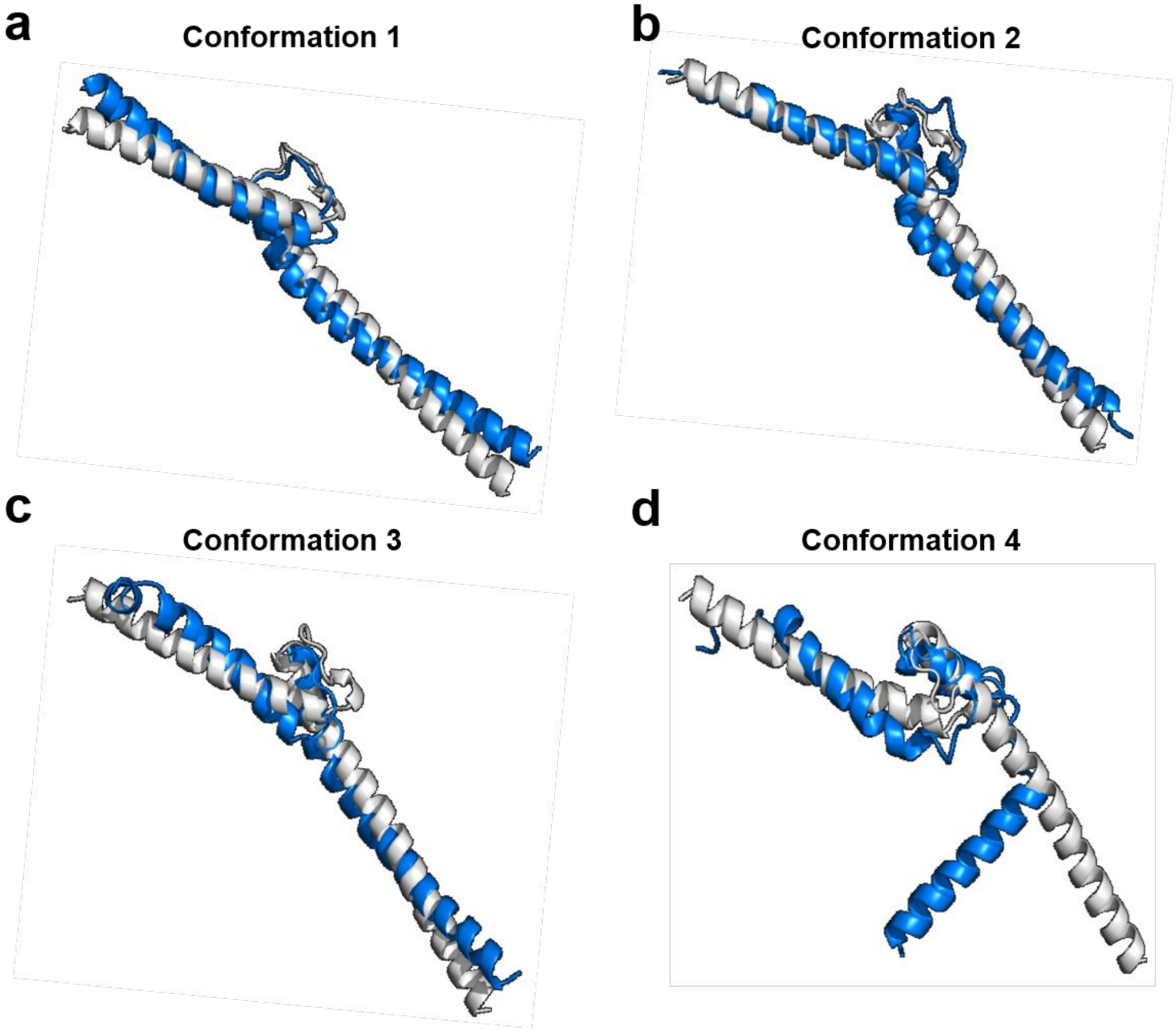

Figure S5. The comparison of the bHLH-Zipper motif of each **(a)** Conformation 1, **(b)** Conformation 2, **(c)** Conformation 3, and **(d)** Conformation 4 with the crystal structure of the bHLH-Zipper motif (PDB ID: 6G6K). The crystal structure is shown in grey while the bHLH-Zipper motif of all four conformations are shown in blue.

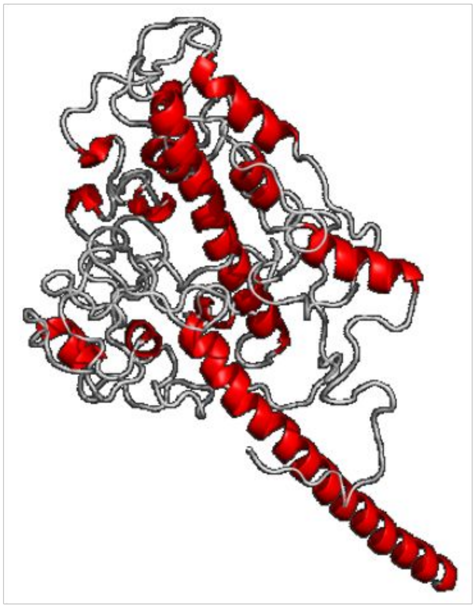

Conformation 1

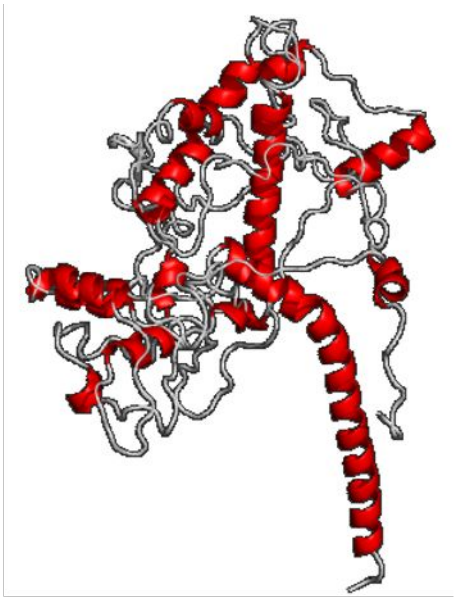

Conformation 2

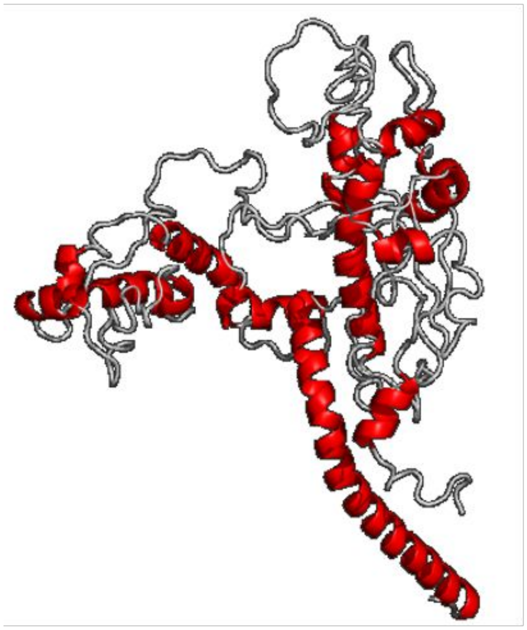

Conformation 3

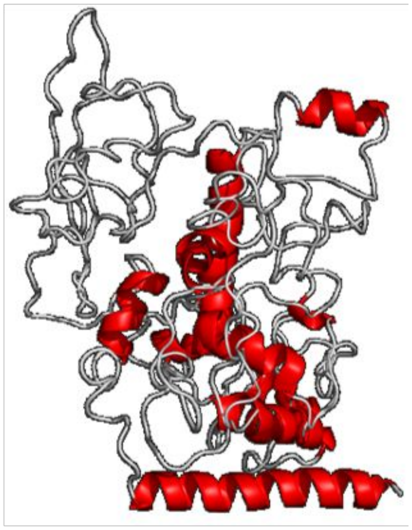

Conformation 4

Figure S6. The number of α-helices formed in each conformation. The α-helices are shown in red.

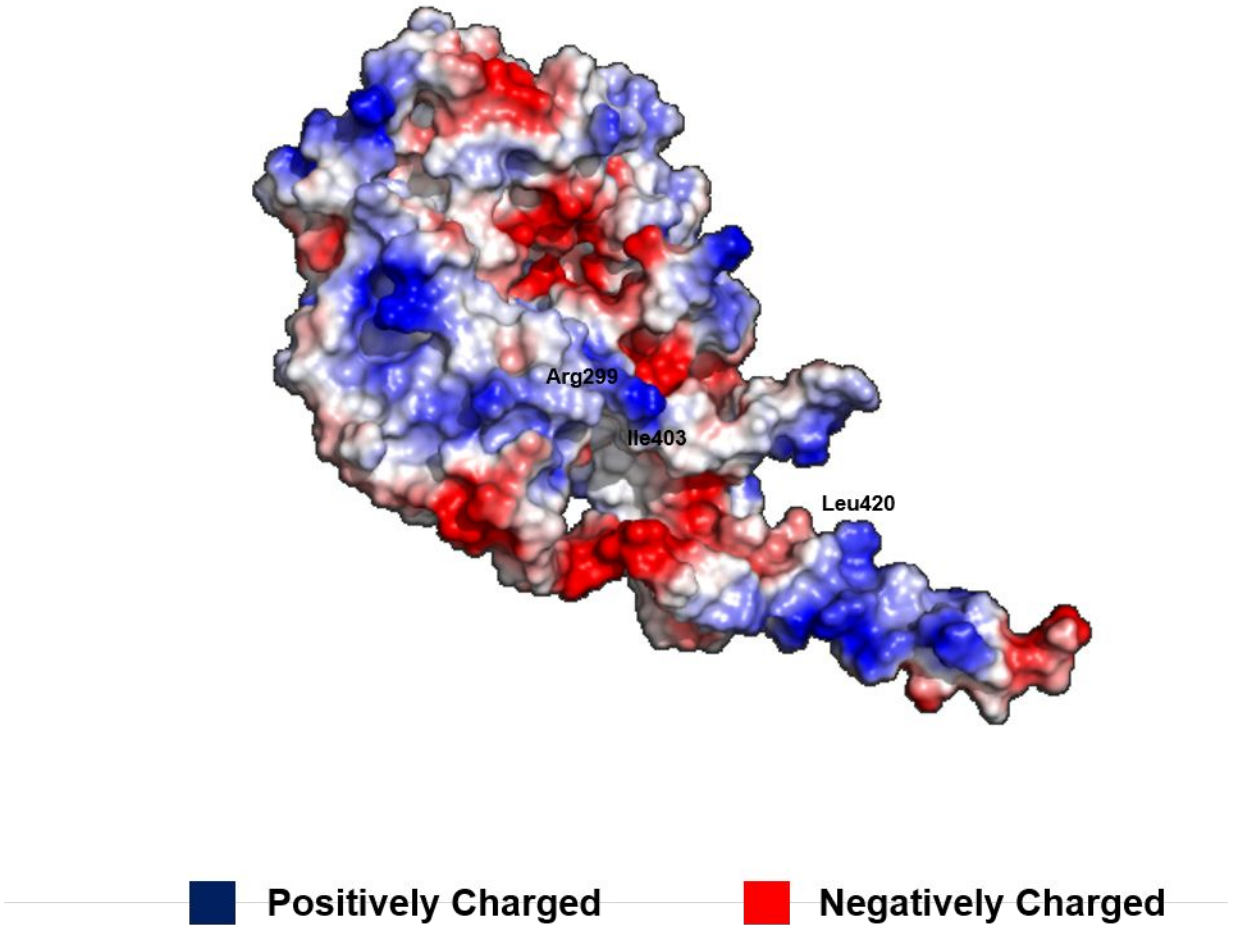

Figure S7. The electrostatic map of c-Myc. The residues that are positively charged are shown in blue and the negatively charged residues are shown in red. The top three residues that show highest contribution towards Max interaction are shown.

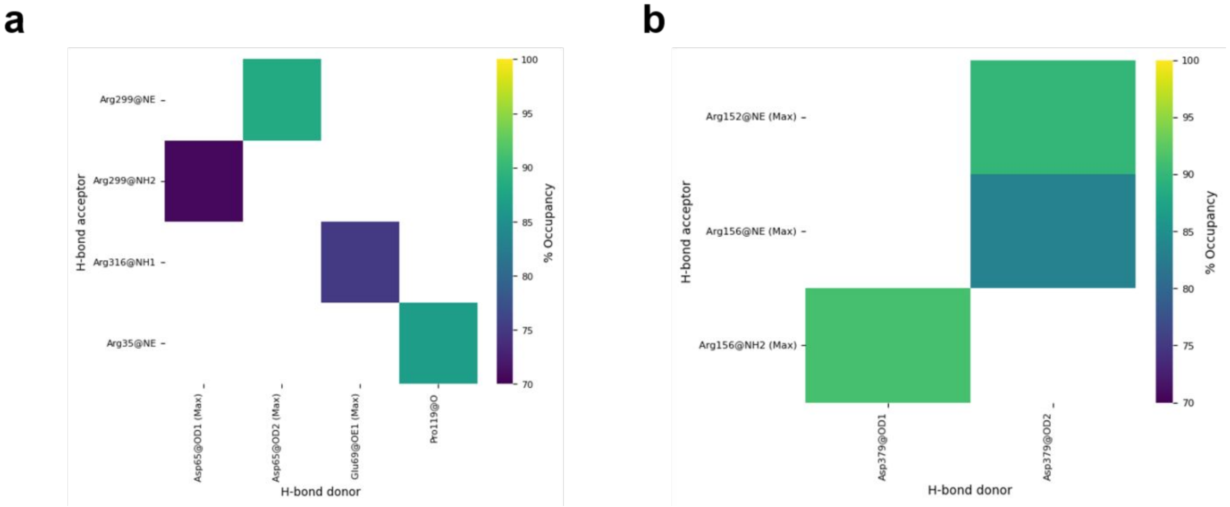

Figure S8. The number of H-bonds formed between c-Myc and Max (intermolecular) throughout the simulation with greater than 70% occupancy in **(a)** replicate 1 and **(b)** replicate 2.
